# Adult Annular Pancreas Presenting With Gastric Outlet Obstruction Following Semaglutide Initiation: A Case Report

**DOI:** 10.7759/cureus.111636

**Published:** 2026-06-27

**Authors:** Fatima Gamaleldin, Milind Raje, Solomon K John

**Affiliations:** 1 College of Medicine, University of Sharjah, Sharjah, ARE; 2 Radiology, NMC Royal Hospital Sharjah, Sharjah, ARE; 3 Hepato-Biliary-Pancreatic, Gastro-Intestinal, and Transplant Surgery, NMC Royal Hospital Sharjah, Sharjah, ARE

**Keywords:** annular pancreas, duodenal stenosis, gastric outlet obstruction, glp-1 receptor agonist, semaglutide

## Abstract

Annular pancreas is a rare congenital anomaly that may remain clinically silent until adulthood. Its symptoms can be masked or exacerbated by glucagon-like peptide-1 receptor agonists (GLP-1 RAs), which delay gastric emptying. We report a 55-year-old male with type 2 diabetes who developed progressive postprandial vomiting and nine-kg weight loss following semaglutide initiation. Imaging revealed a partial annular pancreas causing second-part duodenal stenosis without malignancy. Symptoms persisted despite semaglutide discontinuation, supporting a fixed mechanical obstruction as the primary aetiology. The patient underwent successful Roux-en-Y gastrojejunostomy and cholecystectomy; histopathology confirmed benign fibrotic pancreatic tissue. At follow-up, the patient was symptom-free with nutritional recovery. This case emphasises that severe gastrointestinal symptoms in patients on GLP-1 RAs should prompt evaluation for structural pathology rather than being attributed solely to medication side effects, ensuring timely surgical intervention.

## Introduction

Annular pancreas is a rare congenital anomaly in which pancreatic tissue encircles the duodenum and may remain asymptomatic until adulthood, often presenting as gastric outlet obstruction and mimicking periampullary malignancy [1-6]. Key clinical red flags that should raise suspicion for fixed mechanical obstruction rather than medication-related delayed gastric emptying include persistent vomiting despite drug discontinuation, significant unintentional weight loss, and imaging evidence of duodenal narrowing. Glucagon-like peptide-1 (GLP-1) receptor agonists (RAs) delay gastric emptying by slowing antral contractility and reducing vagal tone, which may amplify symptoms from a pre-existing subclinical structural lesion rather than causing obstruction de novo. Diagnosis requires radiologic and endoscopic evaluation. This challenge is further complicated by GLP-1 RAs such as semaglutide, which can cause nausea, vomiting, early satiety, and delayed gastric emptying and may mask underlying structural disease [7-10]. We report a case in which semaglutide appeared to unmask previously silent duodenal obstruction due to annular pancreas, underscoring the need to exclude mechanical causes before attributing severe symptoms to drug intolerance. This case report has been prepared in line with the Surgical CAse REport (SCARE) 2023 Criteria [11].

## Case presentation

A 55-year-old Arab man with type 2 diabetes mellitus, BMI 30.3 kg/m^2^, and no smoking or alcohol history presented in 2025 with a three-month history of severe progressive postprandial vomiting and an unintentional nine-kg weight loss. His diabetes was managed with subcutaneous semaglutide 0.5 mg once weekly for glycaemic optimisation, with no dose escalation, and he was not taking any other antidiabetic agents or medications known to impair gastric motility, including opioids, anticholinergics, or calcium channel blockers. Symptoms persisted for several weeks after semaglutide discontinuation. He had been entirely asymptomatic from a gastrointestinal standpoint prior to semaglutide initiation. Symptoms worsened within weeks of starting the medication and critically persisted after its discontinuation, arguing against isolated medication intolerance or semaglutide-induced gastroparesis as the primary aetiology, since drug-induced delayed gastric emptying would be expected to resolve following cessation. He denied melena, hematochezia, steatorrhea, abdominal pain, or previous episodes of nausea, vomiting, or early satiety. There was no known family history of pancreatic anomalies, gastrointestinal malformations, or hereditary pancreatitis.

On assessment, pre-operative weight was 87.8 kg, serum albumin was 3.7 g/dL (normal: 3.5-5.0 g/dL), carbohydrate antigen 19-9 (CA 19-9) was 14.95 U/mL (normal: <37 U/mL), and carcinoembryonic antigen (CEA) was <0.5 ng/mL (normal: <2.5 ng/mL in non-smokers). Magnetic resonance imaging demonstrated a partial annular pancreas encircling and causing extrinsic stenosis of the second part of the duodenum (D2), with upstream dilatation of D1, a pancreas divisum variant, and smooth tapering of a mildly dilated common bile duct, without a discrete mass (Figures 1, 2).

**Figure 1:**
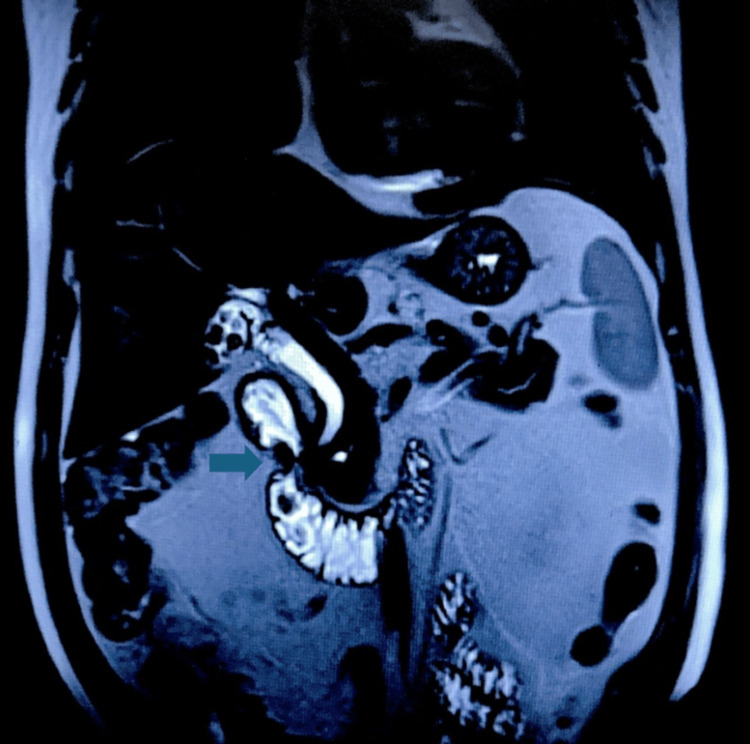
Coronal MRI demonstrating a partial annular pancreas (arrow) encircling the second part of the duodenum (D2) with resultant extrinsic stenosis and upstream dilatation of the first part of the duodenum (D1). A pancreas divisum variant is also noted.

**Figure 2:**
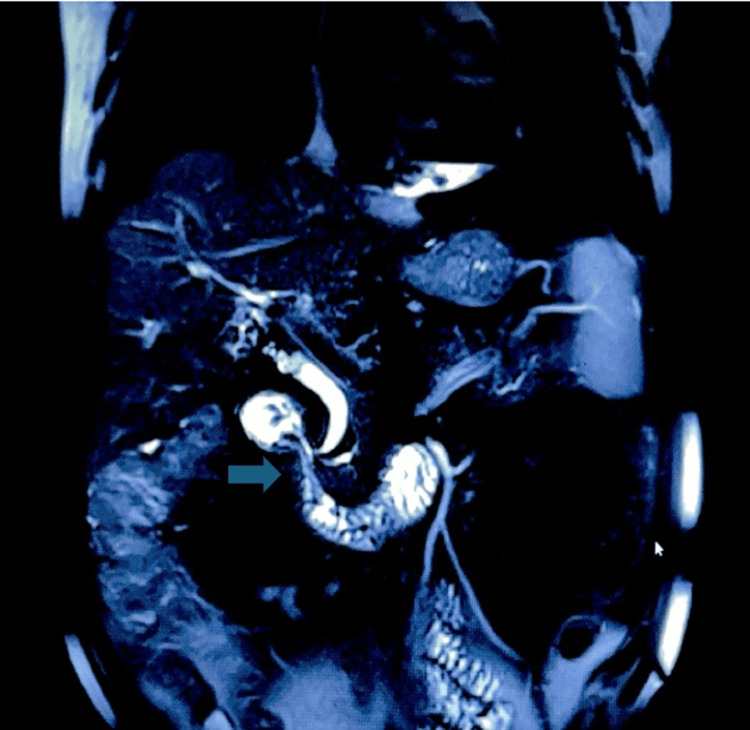
Coronal MRI showing smooth tapering of the mildly dilated common bile duct without evidence of a discrete mass lesion or periductal infiltration, helping to distinguish annular pancreas from periampullary malignancy (arrow).

Magnetic resonance cholangiopancreatography (MRCP) confirmed the absence of an obstructing biliary or pancreatic ductal lesion. Computed tomography corroborated the duodenal stenosis at D2, and the site of narrowing was marked by the blue arrow in Figure 3. Endoscopic ultrasound with duodenal biopsy showed chronic duodenitis only.

**Figure 3:**
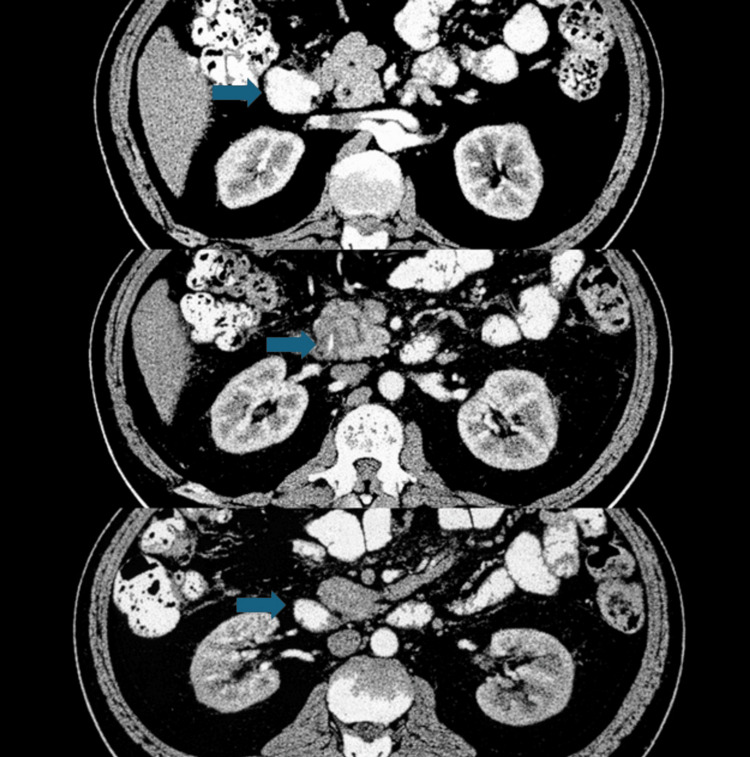
Axial CT images of the abdomen at three sequential levels. The blue arrow indicates the site of duodenal narrowing at the level of the annular pancreatic ring (D2). No discrete mass, lymphadenopathy, or vascular involvement is demonstrated.

The main differential diagnoses included periampullary carcinoma, pancreatic head malignancy, groove pancreatitis, peptic-related duodenal stricture, and GLP-1 RA-induced delayed gastric emptying. Malignancy was considered less likely because cross-sectional imaging showed no discrete mass, lymphadenopathy, or vascular involvement; tumour markers were normal; MRCP showed no obstructing ductal lesion; and endoscopic biopsy revealed only chronic duodenitis, with benignity later confirmed by intraoperative frozen section and final histopathology. Groove pancreatitis was excluded by the absence of a sheet-like fibrous mass in the pancreaticoduodenal groove on MRI. Peptic stricture was unlikely because no ulcer disease was seen on endoscopy. GLP-1 RA-induced gastroparesis was considered, but it could not explain the persistent symptoms after semaglutide discontinuation. Gastric emptying scintigraphy was not performed, which limited formal quantification of delayed gastric emptying versus fixed mechanical obstruction.

An exploratory laparotomy was performed through a right subcostal incision extended to the midline subxiphisternum. Intraoperatively, the first part of the duodenum was markedly dilated, with a cicatrised and partially stenosed distal D2 segment overlaid by pancreatic tissue. No definite mass was identified. A trucut biopsy of the pancreatic tissue overlying D2 was sent for frozen section, which was negative for malignancy, helping avoid pancreaticoduodenectomy. No hepatic metastases, peritoneal nodules, or ascites were seen. The liver appeared fatty.

The gallbladder was distended with stones, and the Calot triangle was inflamed, consistent with symptomatic cholelithiasis, so concurrent cholecystectomy was performed. A Roux-en-Y gastrojejunostomy was fashioned using a retrocolic Roux limb, with a side-to-side jejunojejunostomy created 20 cm from the duodenojejunal flexure and 40 cm distal to the gastrojejunostomy. Insertion of a nasojejunal feeding tube was attempted, but it could not be advanced beyond the stomach, so a nasogastric tube was left in situ. A drain was placed in the right upper quadrant adjacent to the gastrojejunostomy and gallbladder bed. Haemostasis was satisfactory, and there was no evidence of bile or enteric leak before closure. Final histopathology confirmed benign pancreatic acinar tissue with chronic inflammation and fibrosis, without atypia, dysplasia, or malignancy.

Vomiting resolved completely after surgery, and the patient tolerated progressive oral intake without difficulty. He was discharged on pantoprazole and a daily multivitamin, while glycaemic management was continued with empagliflozin, and semaglutide was not restarted. Serial weight measurements demonstrated progressive nutritional recovery, with weight increasing from 83.5 kg to 85.0 kg at follow-up. The postoperative course was uncomplicated, corresponding to Clavien-Dindo Grade 0. Wound healing was unremarkable, and no re-exploration was required. Ongoing surveillance will include periodic clinical review and assessment of nutritional indices, including albumin, vitamin B12, and haematinics, with cross-sectional imaging reserved for clinical indications. The patient expressed satisfaction with the surgical outcome.

## Discussion

Adult annular pancreas presenting with gastric outlet obstruction poses a significant diagnostic challenge, as its clinical and radiological features can closely mimic periampullary malignancy [4-6]. A systematic approach incorporating cross-sectional imaging, MRCP, endoscopic ultrasound, and tumour markers is therefore essential to exclude malignancy before surgical planning. Representative cases from the literature, alongside the present case, are summarised in Table 1, illustrating the breadth of clinical presentations and the consistency of surgical bypass as the definitive management strategy [1-6]. In the absence of a confirmed or strongly suspected malignancy, surgical bypass remains the preferred approach and has demonstrated favourable outcomes across adult case series [1-3,5,6]. When diagnostic uncertainty persists intraoperatively, frozen section biopsy of the pancreatic tissue is a critical adjunct - as demonstrated in this case - allowing safe exclusion of malignancy and thereby avoiding unnecessary pancreaticoduodenectomy with its associated morbidity. In this case, Roux-en-Y gastrojejunostomy was selected over duodenoduodenostomy for several reasons: the significant cicatricial stenosis and marked proximal duodenal dilatation made a tension-free duodenoduodenostomy technically challenging; Roux-en-Y gastrojejunostomy provides durable long-segment diversion well suited to concurrent cholecystectomy; and this configuration may reduce bile reflux gastritis, which is particularly relevant in a patient who may require future pharmacotherapy affecting gastrointestinal motility. Annular division was avoided because incision of the annular ring carries a substantial risk of pancreatic fistula, haemorrhage, and pancreatitis without superior long-term outcomes compared with bypass, and it is not recommended when bypass is technically feasible [5,6].

**Table 1:** Summary of selected representative case reports of adult annular pancreas. GOO: gastric outlet obstruction; GJ: gastrojejunostomy; D2: second part of duodenum; NGT: nasogastric tube; CT: computed tomography; MRI: magnetic resonance imaging; T2DM: type 2 diabetes mellitus; GLP-1 RA: glucagon-like peptide-1 receptor agonist

Study	Patient Characteristics	Presentation	Key Diagnostic Finding	Management	Main Lesson
Ali et al., 2024 [1]	55F, no GLP-1 RA	Six-month intermittent colicky epigastric pain, nonbilious vomiting	CT: annular pancreas with gastric distention	Gastrojejunostomy + jejunojejunostomy	Adult annular pancreas can present with GOO; surgical bypass is effective
Alshiekh et al., 2024 [2]	47F, no GLP-1 RA	Two-year abdominal pain, six-month postprandial vomiting, weight loss	CT: complete pancreatic ring encircling D2	Side-to-side duodenoduodenostomy (open)	Duodenoduodenostomy is an effective alternative to GJ for segmental obstruction
Kryzsko et al., 2024 [3]	49F, no GLP-1 RA	Progressive vomiting and GOO symptoms	CT: duodenal obstruction at annular ring; NGT decompression pre-operatively	Open antecolic side-to-side gastrojejunostomy	Open gastrojejunostomy is safe and effective, with early postoperative recovery
Present case	55M, T2DM, semaglutide	Progressive vomiting, nine-kg weight loss after GLP-1 RA initiation	Partial annular pancreas on MRI/CT; intraoperative frozen section negative for malignancy	Roux-en-Y GJ + cholecystectomy	GLP-1 RAs may unmask subclinical mechanical obstruction; structural causes must be excluded

The case also illustrates the potential for GLP-1 RAs to unmask underlying structural disease. By delaying gastric emptying, semaglutide likely amplified symptoms from a pre-existing subclinical stenosis [7-9]. Post-marketing data have associated GLP-1 RAs with gastroparesis and intestinal obstruction, although causality remains unestablished [10]. Critically, symptom persistence after semaglutide discontinuation supported a fixed mechanical lesion as the main cause. Had the clinician attributed symptoms solely to drug intolerance, progressive malnutrition and acute-on-chronic obstruction may have ensued. This case therefore supports a stepwise, multidisciplinary approach: severe or refractory upper gastrointestinal symptoms in patients receiving GLP-1 RAs should prompt cross-sectional imaging and endoscopy to exclude structural pathology before any drug modification alone [4-6]. The Roux limb was fashioned 40 cm downstream from the gastrojejunostomy to ensure an adequate anti-reflux limb length [12].

The principal limitations of this report include the absence of gastric emptying scintigraphy, which would have allowed formal quantification of GLP-1 RA-related gastric delay as a contributor to obstruction. The unavailability of MRCP source images for formal re-review is also noted, although the radiological report confirmed the absence of obstructing biliary or ductal pathology. Family history of pancreatic or gastrointestinal anomalies was unremarkable, although formal genetic evaluation was not pursued. The short follow-up period precludes assessment of long-term anastomotic patency and nutritional outcomes; ongoing clinical and nutritional surveillance is planned. HbA1c and formal duration of diabetes were not available for documentation, precluding assessment of the contribution of long-standing diabetic autonomic neuropathy to gastric motility impairment.

## Conclusions

In patients receiving GLP-1 RAs, structural causes of gastric outlet obstruction should be excluded before severe gastrointestinal symptoms are attributed to medication alone. Timely imaging, endoscopic assessment, intraoperative frozen section, and a multidisciplinary approach can guide appropriate surgical management and avoid unnecessary radical resection.
